# Local recurrence involving the sternum and ribs following mastectomy and titanium mesh implants for chest wall reconstruction: A case report

**DOI:** 10.3892/ol.2013.1254

**Published:** 2013-03-14

**Authors:** GUOHUA RONG, HUA KANG

**Affiliations:** Department of General Surgery, Xuanwu Hospital, Capital Medical University, Beijing 100053, P.R. China

**Keywords:** local recurrence, breast cancer, chest wall involvement, titanium mesh implant

## Abstract

Approximately 30% of breast carcinoma patients experience local recurrence, which is commonly considered the first sign of treatment failure. Local recurrence involving the deep chest wall may result in thoracic defects and influence normal cardiopulmonary function. Many studies have reported various techniques using different materials for chest wall reconstruction, and titanium mesh has recently received attention as a novel bone substitute. In the present case report, a 46-year-old female who had not yet entered menopause presented for routine follow-up. Her past history was significant for having had a left modified radical mastectomy followed by chemotherapy and tamoxifen treatment for an invasive ductal breast carcinoma. Examination results revealed an invasive ductal carcinoma invading the chest wall. The patient underwent surgical excision and received a titanium mesh implant for chest wall reconstruction. The patient chose to undergo local radiation therapy and endocrine treatment following surgery. Local recurrence of breast cancer involving the deep chest wall is relatively rare. According to the guidelines, surgical excision followed by radiotherapy is the standard treatment and chemotherapy is not recommended. In our case, a titanium mesh was successfully applied for chest wall reconstruction.

## Introduction

Breast cancer is the most common type of cancer in females, accounting for 29% of estimated new cancer cases and 14% of estimated cancer-related mortalities (1). Although breast cancer became curable during the last decade, individuals treated for breast carcinoma remain at risk of local or distant recurrence indefinitely, depending on various factors. Local recurrence is commonly considered a first sign of treatment failure. This may occur contralaterally (on the other breast) or ipsilaterally (on the same side as the initial cancer, either in the remaining breast tissue or on the chest wall). Approximately 30% of patients experience local recurrence of breast cancer (2). Traditional options for the treatment of local recurrence following mastectomy include surgery, radiation therapy (RT), chemotherapy, hormonal therapy or a combination of modalities (3). However, the utility of chemotherapy following locoregional treatment for isolated locoregional recurrence still remains controversial, and has been investigated in a joint study by the International Breast Cancer Study Group (IBSCG), the National Surgical Adjuvant Breast and Bowel Project (NSABP) and the Breast International Group (BIG) (4). The recurrence of tumors invading the sternum and ribs following mastectomy is relatively rare, but treatment problems are frequently encountered (5). The best treatment option is surgical excision; however, the resection of the sternum and ribs occasionally causes defects of the thoracic wall, resulting in secondary complications and influencing the normal cardio-pulmonary function (6). Many studies have reported various techniques using different materials for chest wall reconstruction (7–10) and recently titanium mesh has emerged as a promising and reliable bone substitute (11–13).

In this report, a local recurrence of invasive ductal breast carcinoma which invaded the sternum and several ribs is described. A titanium mesh was used for chest wall construction, which is not often reported in the field of breast cancer treatment. This study was approved by the Ethics Committee of Capital Medical University, Beijing, China Written informed consent was obtained from the patient for the publication of this study.

## Case report

A 46-year-old Asian female who had not entered menopause presented for routine follow-up in April 2012. She had previously undergone a left modified radical mastectomy followed by 8 courses of CMF chemotherapy and tamoxifen treatment for 3 years, for an estrogen receptor (ER) (+++)-positive, progesterone receptor (PR) (+++)-positive, T1N0M0 invasive ductal breast carcinoma. On routine follow-up with clinical and radiological examination the patient was found to be free of any recurrence.

**Figure 1:**
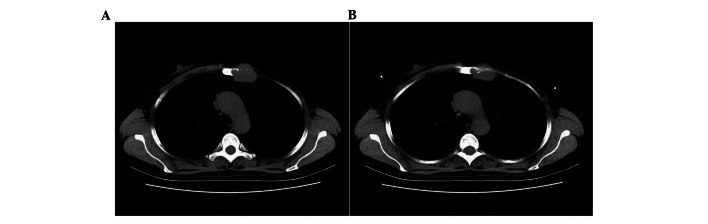
Chest computed tomography (CT) images. (A and B) reveal bone destruction of the left sternum and a 3.4×3.5-cm mass that intruded the left chest cavity.

**Figure 2:**
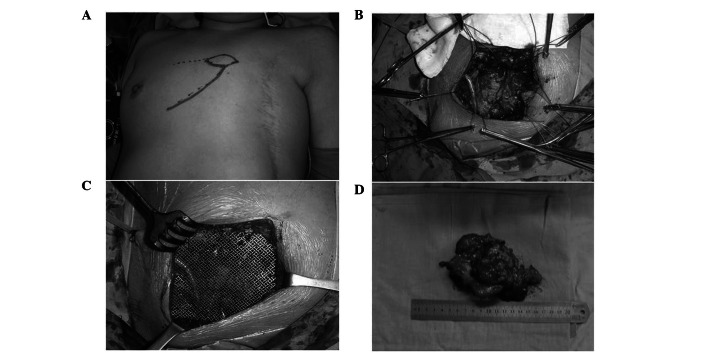
Implantation of titanium mesh during the surgery. (A) Surgical markings made on the skin. (B) Removal of the partial sternum and ribs. (C) Fixation of the titanium mesh. (D) The resected tumor mass.

Physical examination revealed a 2×2-cm nodule in the location of the mastectomy scar that was palpable and fixed to the underlying tissue. Subsequent ultrasound demonstrated a 3-cm hypoechoic nodule that corresponded to the mammographic abnormality. Positron emission tomography (PET) showed no evidence of distant metastases, while chest computed tomography (CT) identified bone destruction of the left sternum and a 3.4×3.5-cm mass which intruded into the left chest cavity (Fig. 1). Ultrasound-guided core needle biopsy of the mass revealed an invasive ductal carcinoma that was ER (+, 90%) and PR (+, 80%)-positive. Considering the local recurrence of breast carcinoma invading the chest cavity along with bone destruction, the patient underwent extended lumpectomy with partial excision of the sternum and ribs (second, third and fourth) and a titanium mesh was implanted for chest wall reconstruction (Fig. 2). The final pathology showed invasive ductal carcinoma invading the adjacent ribs and soft tissues, and all margins were negative by >10 mm. Immunostaining for ER and PR were both positive, while the expression of human growth factor receptor 2 (HER-2) was negative. Following the guidelines of the National Comprehensive Cancer Network (NCCN), the patient chose to undergo local extended-field RT and endocrine treatment following surgery.

## Discussion

Local recurrence of breast cancer may occur as an isolated event or concomitantly with systemic spread of disease. Isolated local recurrences following breast conserving therapy are highly curable by salvage mastectomy. However, local recurrence after mastectomy may present as a first sign of widespread metastatic disease. Therefore, patients suffering local recurrence following mastectomy should undergo a workup for metastatic disease. In the absence of distant metastases, surgical excision followed by RT to the involved chest wall and regional lymphatics is the standard treatment approach (3,14). Fodor *et al* reported that certain factors such as disease-free interval, initial tumor and lymph node stage, patient age, extent and histology of the recurrent tumor are associated with the risk of cause-specific mortality following local recurrence in patients with early-stage invasive breast cancer (15).

Chest wall breast cancer recurrence following mastectomy is considered a difficult disease to treat, and usually involves multiple cutaneous and subcutaneous tumor nodules. Recurrences invading deeper chest wall structures similar to the present case are far less prevalent (16). Many studies have shown that postmastectomy RT is beneficial in reducing chest wall recurrence, particularly in cases with larger tumors and axillary lymph node involvement (5,17). Surgical excision followed by RT has been acknowledged as the best treatment option. This was found to be related with prognosis in cases where there is no metastasis and when the recurrence develops more than 24 months after mastectomy and patients are initially node-negative (6). According to the large randomized trials being performed by the International Breast Cancer Study Group (IBCSG), the National Surgical Adjuvant Breast and Bowel Project (NSABP) and the Breast International Group (BIG), the use of chemotherapy following radical local treatment of locoregional relapse remains controversial (4). In the present case, the oncologists did not recommend chemotherapy for postoperative treatment considering the recurrence of invasive ductal breast cancer after 8 complete courses of CMF chemotherapy.

Furthermore, local recurrence involving the deep chest wall results can result in thoracic defects generated by surgery, and chest wall reconstruction is sometimes required (18). Large anterior and lateral resections render intrathoracic structures vulnerable to external impact and necessitate rigid reconstructions. These include a number of techniques using alloplastic materials such as methyl methacrylate-based customized plates, neo-ribs, osteosynthesis systems or a dedicated prosthesis (19). Currently, as a novel bone substitute for reconstruction, titanium mesh merits attention. Titanium mesh has been acknowledged as highly versatile and easy to implement. It lacks the problems associated with other materials and is substantial enough to provide the support required. In particular, titanium mesh does not interfere with postoperative imaging such as X-ray, CT and magnetic resonance imaging (MRI). Additionally, it has been concluded that the dosimetric impact of titanium mesh is minimal and does not require modification in radiotherapy treatment parameters (20). Kim *et al* found that a coronal flap with frontal craniotomy and orbital roof reconstruction using titanium mesh provided good functional and cosmetic results (21). Fabre *et al* reported that following sternectomy for cancer, reconstruction with a titanium rib bridge system had low morbidity and permitted a rapid return to baseline pulmonary mechanics (13). In the present case, the titanium mesh was used for chest wall reconstruction as the left sternum and three ribs were resected. The surgery went smoothly and the prognosis of the patient and treatment outcome will be followed up.

In summary, local recurrence of breast cancer involving deep chest walls is relatively rare. According to the guidelines, surgical excision followed by RT is the standard treatment. The beneficial effect of chemotherapy after radical local treatment of locoregional relapse remains contraversial, and for this reason chemotherapy is not recommended. Recently, titanium mesh has emerged as a promising and reliable bone substitute, and was applied in the present case. The prognosis of the patient will be followed up and the effectiveness of the titanium mesh implant for chest wall reconstruction will be evaluated.
